# On an Atrophy of the Testicles *Observed in Egypt*

**Published:** 1804-12-01

**Authors:** 

**Affiliations:** Chief Surgeon of the Army


### On an Atrophy of the Testicles *observed in Egypt; by* Mr. Larrey, *Chief Surgeon of the Army*.

SEVERAL soldiers of the Army of Egypt having complained of the total abolition of the testicles, without any previous venereal disease; Mr. Larrey, surprized at this phenomenon, endeavoured to discover the cause, and to observe the progress of this singular malady; the results of which are as follow.

Generally one of the testicles gradually lost its sensibility, became soft, diminished in size, and seemed to be exsiccated. The patient perceives nothing of this destruction, which proceeds insensibly, till the testicle being reduced to a very small size, is found near the annulus abdominalis, in form and size of a white bean; then it becomes indolent, and grows hard, and the spermatic chord decreases likewise, and participates of this disease.

When both testicles are affected, the patient is deprived of his generative faculty, which he observes by the total absence of carnal desires and amorous sensations, and by the laxity of the genitals; and this, the author has found to be the case with all that experienced this malady, and which influences likewise the whole system, as it grows also weak; the lower extremities emaciate, and the pace becomes waddling; the countenance is changed, the beard grows thin, the stomach becomes weak, the digestion is impaired, and the mental faculties deranged. Several soldiers became invalids in consequence of this disease.

Mr. Larrey ascribes this malady chiefly to the intense heat of the Egyptian climate, which by relaxing the texture ture of the testicle, disposes it to that sort of dissolution; the most fluid parts of this organ go off by transpiration, and another portion is absorbed by the lymphatic system, and brought within the course of circulation. The parenchyma of the vessels, which resist these effects, shrinks, the tubes contract and dry up, the whole mass of the testicle loses more or less in its compass, and becomes emaciated. To this, as a principal cause, may be added, the fatigues of war, and the frequent wants which the troops experienced, but more particularly the use of the spirit from dates, which the inhabitants, in order to make it stronger and more palatable, draw off with the fruits of several solanaceous plants, as the pseudocapsicum, and the common capsicum, both which serve as spices: and, perhaps, says Mr. Larrey, they know it by experience or tradition, that these substances diminish the nervous sensibility, which much easier developes itself in hot climates, and becomes more susceptible of too great a mobility. The author takes an opportunity to remark, that he has likewise observed the paralytic effects of the bella-donna on the organ of sight; and he cautions particularly against employing solanaceous remedies in hot climates. When the atrophy of the testicles is perfect or complete, the art offers no remedy; but when it is only commencing, the disagreeable consequences may be prevented by cold baths, by dry fictions of the body, by urtication applied to the breech, by stomachic remedies, and a full diet.

In order to be secured from this accident, it is advisable to avoid the use of spirituous liquors, and particularly of the spirit drawn from dates by the Egyptians; further, to wear a straight suspensorium, to wash the body frequently with fresh water and vinegar, and to abstain from the sexual intercourse.

